# Regulation of the Coral-Associated Bacteria and Symbiodiniaceae in *Acropora valida* Under Ocean Acidification

**DOI:** 10.3389/fmicb.2021.767174

**Published:** 2021-12-17

**Authors:** Ruiqi Ge, Jiayuan Liang, Kefu Yu, Biao Chen, Xiaopeng Yu, Chuanqi Deng, Jinni Chen, Yongqian Xu, Liangyun Qin

**Affiliations:** ^1^Guangxi Laboratory on the Study of Coral Reefs in the South China Sea, Coral Reef Research Center of China, School of Marine Sciences, Guangxi University, Nanning, China; ^2^Southern Marine Science and Engineering Guangdong Laboratory (Zhuhai), Zhuhai, China

**Keywords:** Symbiodiniaceae, coral-associated bacteria, coral holobiont, ocean acidification, photosynthetic efficiency

## Abstract

Ocean acidification is one of many stressors that coral reef ecosystems are currently contending with. Thus, understanding the response of key symbiotic microbes to ocean acidification is of great significance for understanding the adaptation mechanism and development trend of coral holobionts. Here, high-throughput sequencing technology was employed to investigate the coral-associated bacteria and Symbiodiniaceae of the ecologically important coral *Acropora valida* exposed to different pH gradients. After 30 days of acclimatization, we set four acidification gradients (pH 8.2, 7.8, 7.4, and 7.2, respectively), and each pH condition was applied for 10 days, with the whole experiment lasting for 70 days. Although the Symbiodiniaceae density decreased significantly, the coral did not appear to be bleached, and the real-time photosynthetic rate did not change significantly, indicating that *A. valida* has strong tolerance to acidification. Moreover, the Symbiodiniaceae community composition was hardly affected by ocean acidification, with the C1 subclade (*Cladocopium goreaui*) being dominant among the Symbiodiniaceae dominant types. The relative abundance of the Symbiodiniaceae background types was significantly higher at pH 7.2, indicating that ocean acidification might increase the stability of the community composition by regulating the Symbiodiniaceae rare biosphere. Furthermore, the stable symbiosis between the C1 subclade and coral host may contribute to the stability of the real-time photosynthetic efficiency. Finally, concerning the coral-associated bacteria, the stable symbiosis between *Endozoicomonas* and coral host is likely to help them adapt to ocean acidification. The significant increase in the relative abundance of Cyanobacteria at pH 7.2 may also compensate for the photosynthesis efficiency of a coral holobiont. In summary, this study suggests that the combined response of key symbiotic microbes helps the whole coral host resist the threats of ocean acidification.

## Introduction

Since the Industrial Revolution, the concentration of atmospheric CO_2_ has continuously increased. As the most significant carbon pool on the earth, the ocean has continuously absorbed CO_2_ from the atmosphere, causing the pH of the surface seawater to drop and consequently, promoting ocean acidification, which is one of the biggest threats to the global coral reef ecosystem (Hoegh-Guldberg et al., 2007). Ocean acidification affects the photosynthesis, calcification, growth, and survival of coral reef (Albright and Langdon, 2011). Moreover, it also reduces the calcification of reef-building corals by changing the chemical composition of seawater carbonates (Leclercq et al., 2002; Kroeker et al., 2010), and changes in CO_2_ content in seawater affect coral photosynthesis to varying degrees (Comeau et al., 2017; Kenkel et al., 2018). Ocean acidification can also lead to changes in the community structure of coral-associated bacteria, thereby affecting the physiological characteristics of the coral host (Thurber et al., 2009; Meron et al., 2011). The fourth report of the Intergovernmental Panel on Climate Change (IPCC) in 2007 focused on the adverse effects of ocean acidification on corals and other calcareous organisms (Mingle, 2020). The report titled “The ocean and cryosphere in a changing climate,” released by the IPCC in 2019, further pointed out that ocean acidification and other environmental pressures have impacted the distribution and abundance of marine organisms (Mingle, 2020). Hence, if CO_2_ emissions continue to increase, the existing coral reef ecosystems will face survival problems by the end of the 21st century (Ricke et al., 2013).

As a mutual symbiont assemblage, coral holobiont includes Symbiodiniaceae, bacteria, fungi, archaea, and other microorganisms (Bourne et al., 2016; LaJeunesse et al., 2018). Symbiodiniaceae is an essential part of the coral holobiont, as they fix carbon through photosynthesis and provide up to 95% of the energy requirements of the host coral (Muscatine and Cernichiari, 1969; Steen and Muscatine, 1984; Muscatine et al., 1989; Rädecker et al., 2015). Coral-associated bacteria are the most basic and active part of the coral holobiont. They play an essential role in the circulation and transportation of nutrients including carbon, nitrogen, sulfur, phosphate, and trace metal elements (Bourne et al., 2016). Noteworthy, the diversity and distribution characteristics of coral holobionts are easily affected by changes in the surrounding environment (Ben-Haim et al., 2003; Bourne and Munn, 2005; Bourne et al., 2008). Thus, rising sea temperature, ocean acidification, and nutrient changes affect the composition of coral holobionts, thereby affecting the health of corals.

Several studies addressed the impact of high-temperature stress on coral holobionts (Thurber et al., 2009; Yu et al., 2020, 2021), whereas relatively few studies investigated the impact of ocean acidification on coral holobionts. Furthermore, the research on coral holobiont by ocean acidification has mainly focused on bacteria, which demonstrated that ocean acidification affects the community composition of adult coral-associated bacteria (Meron et al., 2011; Morrow et al., 2015; O’Brien et al., 2016; Meunier et al., 2021), which will further affect the acidification tolerance of the coral (Liang et al., 2017; Torda et al., 2017). Meron et al. (2011) found that the diversity of *Acropora eurystoma*-associated bacteria significantly increased when seawater pH dropped from 8.2 to 7.3. The number of bacteria related to disease and stress, such as *Rhodobacter* sp., *Bacteroidetes* sp., and Proteobacteria, increased. The study by Thurber et al. (2009) also highlighted that the diversity of *Porites compressa*-associated bacteria fluctuates sharply under a low pH environment (pH 6.7). Including the emergence of viral lysogen-related stress response genes, the relatively rare *Bacteroidetes* increased by 14 times at pH 6.7 and *Crenarchaeota* increased by three times. These studies have shown that ocean acidification can lead to changes of the community composition in the coral-associated bacteria. However, the effects of ocean acidification on Symbiodiniaceae remain poorly understood (Goiran et al., 1996; Reynaud et al., 2003). Previous studies showed that ocean acidification decreases Symbiodiniaceae density and affects its photosynthetic efficiency (Krief et al., 2010; Comeau et al., 2017).

Herein, a laboratory-based simulation experiment was used to analyze the community dynamics and physiological changes of key symbiotic members (Symbiodiniaceae and bacteria) in an environmentally sensitive branch coral (*A. valida*) exposed to seawater with different pH values (pH 8.2, 7.8, 7.4, and 7.2). This study aimed to reveal the changes in the community composition of coral-associated bacteria and Symbiodiniaceae under ocean acidification and provide a more comprehensive understanding of their impact on coral reef ecosystems.

## Materials and Methods

### Coral Collection

*Acropora valida* in good growth conditions was collected from the coral communities of Weizhou Island in the Beibu Gulf at a depth of 6 m, with an individual size of approximately 15 cm in diameter. A total of five coral colonies were collected, each separated by a distance greater than 2 m, and were quickly transferred to a foam box filled with seawater (26°C). The samples were transported to the Coral Reef Research Center of Guangxi University within 24 h. Then, each coral colony was cut into four nubbins on average, and they were acclimatized in an aquarium tank for 1 month. The domestication conditions included the use of a breeding tank with a size of 600 mm × 600 mm × 600 mm (length × width × height) to maintain a stable water environment with the following characteristics: water flow, 6,500 L/h; color temperature, 15,000 K (daily daytime 6:00–18:00, given 12 h of light); salinity, 35 g/L, pH 7.5–8.0; Ca^2+^ concentration, 380–450 ppm; Mg^2+^ concentration, 1,200–1,320 ppm; and water temperature, 26°C. During the entire transportation process, the coral samples were transported indoors within 24 h in an incubator filled with seawater, with little disturbance.

### Experimental Setup

After 30 days of cultivation, a new growing point appeared on top of the coral branch, indicating that the individual coral was adapted to the environment of the experimental aquaculture tank and that acidification experiments could be performed. The controlled variable method was used, with pH as a single variable, to keep other water quality parameters stable during the experiment. The ocean acidification simulation system (OA-simulated system, OASys) designed by Zheng et al. (2018), which adjusts the pH of the water by controlling the high-purity CO_2_ injected into the seawater tank, was used to adjust the pH of the experimental water. A pole transducer was used to detect the pH value (± 0.01 pH unit), which was set to four pH gradients (pH 8.2, 7.8, 7.4, and 7.2). Given that the pH of the water measured before acidification fluctuated at a pH range of 8.1–8.3 between daytime and nighttime, a pH of 8.2 was set as the normal seawater pH (control group), and each pH gradient was maintained for 10 days. Five biological replicate colonies were collected for DNA extraction and Symbiodiniaceae density measurement on the last day of each pH gradient. The extracted DNA samples were used for subsequent high-throughput sequencing.

### Chlorophyll Fluorescence Detection

To detect the influence of environmental factors on the photosynthetic efficiency of coral holobionts, the ultra-portable modulated chlorophyll fluorometer Mini-PAM (Walz Heinz GmbH, Effeltrich, Germany) was used to measure the chlorophyll fluorescence parameters. When measuring, the Mini-PAM optical fiber probe was placed at a vertical distance of approximately 2 mm from the coral surface. The effective optical path of the optical fiber was 5.5 mm, and the electrical signal damping and gain were set to 1 and 2, respectively. Under the daylight simulation of each pH gradient, the real-time photosynthetic efficiency, computed as (*F*_m_′–*F*_t_)/*F*_m_′ (*F*_t_ means real-time fluorescence), was measured at the same time to ensure that the measurement time of each pH gradient was the same.

### Symbiodiniaceae Density and Community Composition Analysis

To determine the Symbiodiniaceae density, a Waterpik instrument equipped with sterile seawater was used to rinse the coral surface thoroughly. Then, the rinsing fluid was collected, its volume was recorded with a graduated cylinder, and 4 ml was centrifuged at 4°C, 4,000 rpm for 5 min. The supernatant was removed, and 200 μl of 4% formaldehyde was added to fix the Symbiodiniaceae for later analysis (Shu et al., 2011; Qin et al., 2019). The Symbiodiniaceae fixed on a hemocytometer were observed and counted under an optical microscope. The surface area of the coral tissue was determined according to the correlation between the weight of the foil paper and surface area (Shu et al., 2011).

The Symbiodiniaceae community composition analysis was performed according to the following steps (Chen et al., 2020): Sterile surgical scissors were used to cut the coral samples (about 90 mg) containing the coral tissue, skeleton, and mucus, with five parallel samples at each pH gradient; (2) the Marine Animal Genomic DNA Extraction Kit (TIANGEN, DP324) and Plant Genomic DNA Kit (OMEGA, D3485-2) were used to extract the DNA of coral, and coral-associated bacteria and Symbiodiniaceae, respectively. Mixed animal DNA samples and plant DNA samples, and screened for quality and purity; (3) using forward ITS2 (5'-GAATTGCAGAACTCCGTG-3') and reverse ITS2 (5'-GGGATCCATATGCTTAAGTTCAGCGGGT-3') as primers, PCR amplification was performed on the rDNA Symbiodiniaceae ITS2 region. The reaction was performed in a 20 μl system (TransGen® AP221-02; TransGen Biotech, Beijing, China): 4 μl of 5× Fast *Pfu* buffer, 2 μl of 2.5 mM deoxyribonucleotide triphosphates (dNTPs), 0.8 μl of the forward primer (5 μM), 0.8 μl of the reverse primer (5 μM), 0.4 μl of Fast *Pfu* Polymerase, 10 ng of the DNA template, and ddH_2_O to adjust the final volume (20 μl). The PCR amplification program was: 94°C pre-denaturation for 3 min, and 35 cycles (denaturation at 94°C for 30 s, annealing at 55°C for 30 s, and extension at 72°C for 45 s), and extension at 72°C for 10 min; and (4) the duplicate samples were merged and were submitted to Biomarker Technologies Co., Ltd. (Beijing, China) for sequencing on the Illumina MiSeq (PE 2 × 300) platform (Illumina, San Diego, CA, United States).

Trimmomatic software was used to perform quality control on the Illumina MiSeq platform (Bolger et al., 2014). Reads were truncated at an average quality score < 20 to ensure high-quality reads for subsequent analysis. Used the combined data to obtain a full-length ITS2 rDNA fragment (Zhang et al., 2014), trimmed the read quality, and used Mothur (version 1.34.4) to check the chimeras. Cutadapt (version 1.1) was used to trim the reverse and forward primer sequences (Ziegler et al., 2017). Since the previous ITS2 database contained some repetitive sequences, several published databases were collected (Franklin et al., 2012; Arif et al., 2014; Tong et al., 2017; Ziegler et al., 2017; Chen et al., 2019), and BLASTn was used to compare all sequences with those stored in the ITS2 database (Tong et al., 2017). In addition, to query ITS2 data at different resolutions and facilitate comparison with the results of denaturing gradient gel electrophoresis, to avoid the interference of intragenomic variation, the number of different ITS2 sequences that were present at a minimum cutoff ≥ 1% in at least one of the 20 samples was evaluated (Ziegler et al., 2017).

### Bacterial Community Analysis

Using the total genomic DNA sample as a template, the forward primer 27F [5'-AGAGTTTGATC (C/A) TGGCTCAG-3'] and the reverse primer 1492R [5'-TACGG(C/T) TACCTTGTTACGAC-3'] were used for the PCR amplification of the full-length of the bacterial *16S* rRNA (ABI GeneAmp® 9700; Thermo Fisher Scientific, Waltham, MA, United States; Singer et al., 2016). The PCR expansion reaction was performed in a 20 μl system: 4.5 μl of 5× Fast *Pfu* buffer, 2.5 μl of 2.5 mM dNTPs, 0.6 μl of the forward primer (5 μM), 0.6 μl of the reverse primer (5 μM), 0.6 μl of Fast *Pfu* polymerase, 0.3 μl of BSA, 10 ng of the template DNA, and ddH_2_O to adjust the final volume (20 μl). The PCR amplification program was: 95°C pre-denaturation for 5 min and 30 cycles (denaturation at 95°C for 30 s, annealing at 55°C for 30 s, and extension at 72°C for 90 s), and extension at 72°C for 7 min. For the gel recovery and purification, elution, detection, and quantification of the PCR products, 2% agarose gel electrophoresis, AxyPrep DNA kit, Tris-HCl, 2% agarose electrophoresis, and QuantiFluorTM-ST fluorescence quantification system (Promega, Madison, WI, United States) were used, respectively. The duplicate samples were merged and used for sequencing (BioMarker, Beijing, China) on the Illumina MiSeq (PE 2 × 300) platform.

The sequence was divided into Operational taxonomic units (OTU) at a similarity level of 97%, and the PE reads were spliced, merged, quality controlled, and filtered with reference to the Ribosomal database project used by Liang et al. (2017). The composition and number of bacterial communities in different taxonomic levels (phyla and genus) of each sample were obtained through OTU cluster analysis and species taxonomy analysis. Mothur (Schloss et al., 2011) was used to perform alpha diversity index analysis on sample clustering results to obtain single sample diversity (alpha diversity) through Good’s species coverage (Coverage), community richness (Ace), and community diversity (Shannon) index, reflecting the coverage, abundance, and diversity of the microbial communities. Qiime (Kuczynski et al., 2011) was used to calculate the beta diversity distance matrix. Vegan software performed principal coordinates analysis (PCoA) and revealed the structure of bacterial communities in different groups, and PERMANOVA was used to test the significant differences in bacterial community structure between groups. The linear discriminant analysis effect size (LEfSe) method was used to identify shifts in the abundance of bacterial OTUs among different pH gradients. The significance threshold was set to LDA value > 4.0 and *p* < 0.05 (Segata et al., 2011).

### Statistical Analysis

The SPSS Statistics 19 (IBM Corp., Armonk, NY, United States) was used to perform one-way ANOVA on the real-time photosynthetic efficiency data of the experimental gradient and the Symbiodiniaceae density data of each pH gradient, and Duncan was used for the *post hoc* test. The Kruskal-Wallis test was used to analyze the significant difference in Symbiodiniaceae distribution and bacterial community composition in each pH gradient.

## Results

### Apparent Morphological Changes of *A. valida*

**Figure 1:**
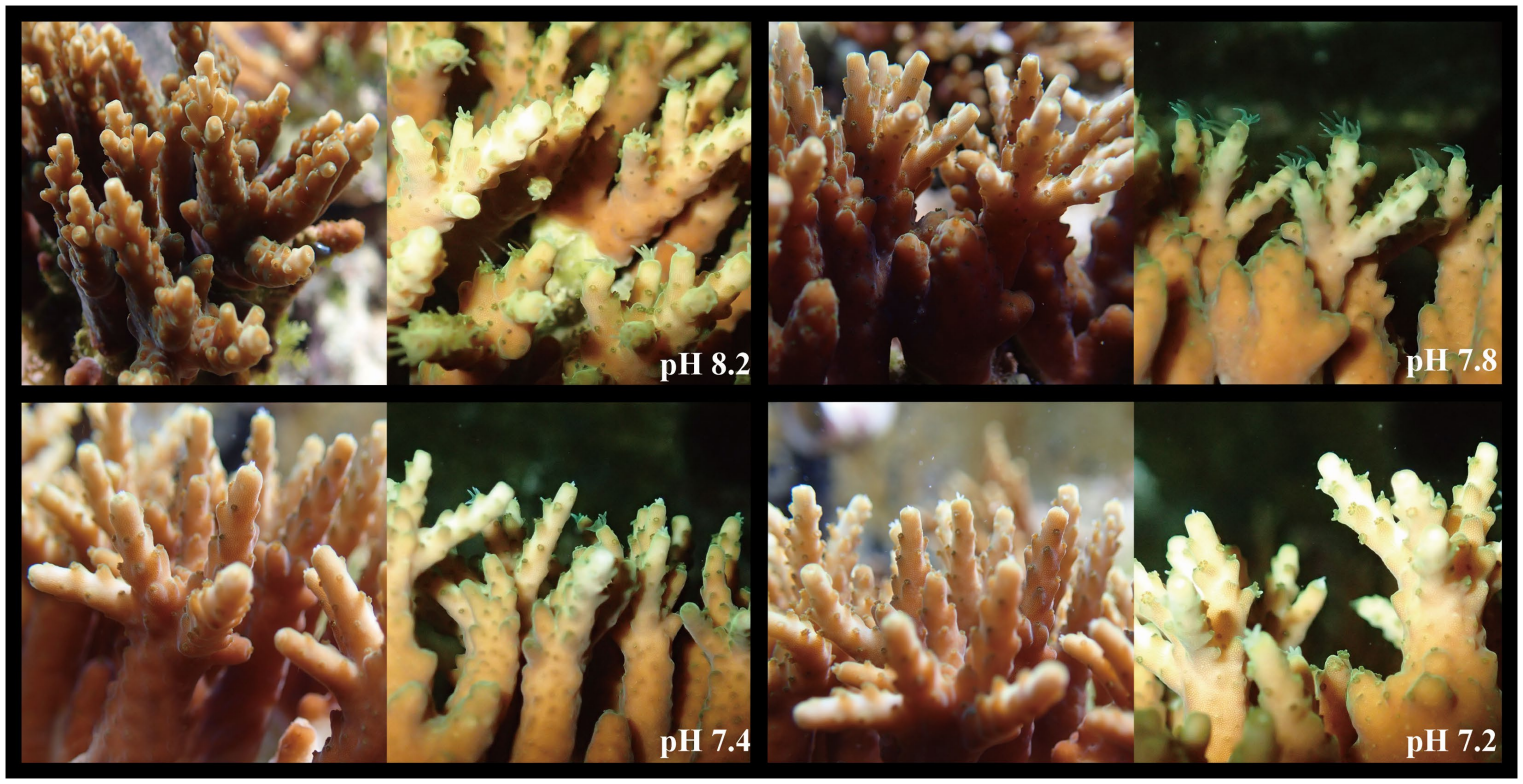
Changes of apparent coral morphology under different pH gradients (two photos for each pH gradient, the left side were taken in simulated daylight, and the right side were taken at night).

As the pH of seawater decreased, the apparent morphology of *A. valida* changed, as visible to the naked eye (Figure 1). At pH 8.2 and 8.0, the color of *A. valida* was dark brown, and its tentacles were freely stretched. When the pH turned to 7.4–7.8, the color of *A. valida* became light brown, and when the pH value became lower (pH 7.2), the color of the coral did not change further, and the coral tentacles could still stretch freely and actively. *Acropora valida* did not appear to be bleached during the experiment when the seawater was acidified from pH 8.2 to 7.2, and holobionts thus appear to have a strong acid resistance.

### Changes in Symbiodiniaceae Density and Real-Time Photosynthetic Efficiency

**Figure 2:**
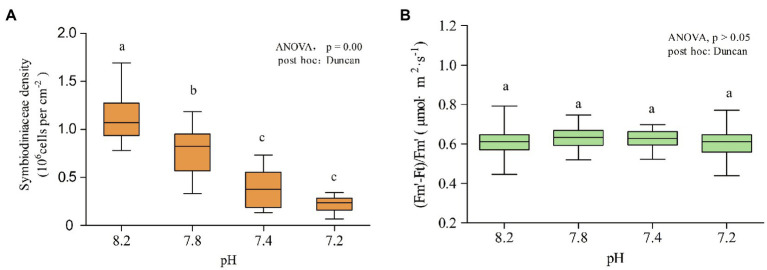
Symbiodiniaceae density and chlorophyll fluorescence at different pH gradients. Each pH condition lasted 10 days. **(A)** Density of Symbiodiniaceae. **(B)** (*F*_m_′-*F*_t_)/*F*_m_′. Lower case letters (a, b, c) denote differences by Duncan *post hoc* test.

As shown in Figure 2A, the density of Symbiodiniaceae changed significantly with the decrease in seawater pH (ANOVA, *F* = 21.325, *p* < 0.005, *post hoc*: Duncan). When the pH of the seawater was 8.2, the average density of Symbiodiniaceae was 1.15 ± 0.50 × 10^6^ cells cm^−2^. At pH 7.8, the Symbiodiniaceae density significantly decreased to 0.77 ± 0.46 × 10^6^ cells cm^−2^. At pH 7.4, the Symbiodiniaceae density continued to drop to 0.38 ± 0.42 × 10^6^ cells cm^−2^ and at pH 7.2 was of 0.22 ± 0.16 × 10^6^ cells cm^−2^, which was only 19% of the density at pH 8.2. Thus, the density of Symbiodiniaceae in *A. valida* fluctuated significantly under the influence of ocean acidification. As the pH gradient dropped, the density of Symbiodiniaceae in the corals decreased significantly. However, as shown in Figure 2B, the real-time photosynthetic efficiency value measured at pH 7.8 was the highest, with an average value of 0.6296 μmol·m^−2^·s^−1^, and the lowest was measured at pH 7.2, with an average value of 0.6050 μmol·m^−2^·s^−1^, showing that acidification had no significant effect on the real-time photosynthetic efficiency of coral (ANOVA, *F* = 1.217, *p* = 0.302, *post hoc*: Duncan).

### Composition of Symbiodiniaceae

**Figure 3:**
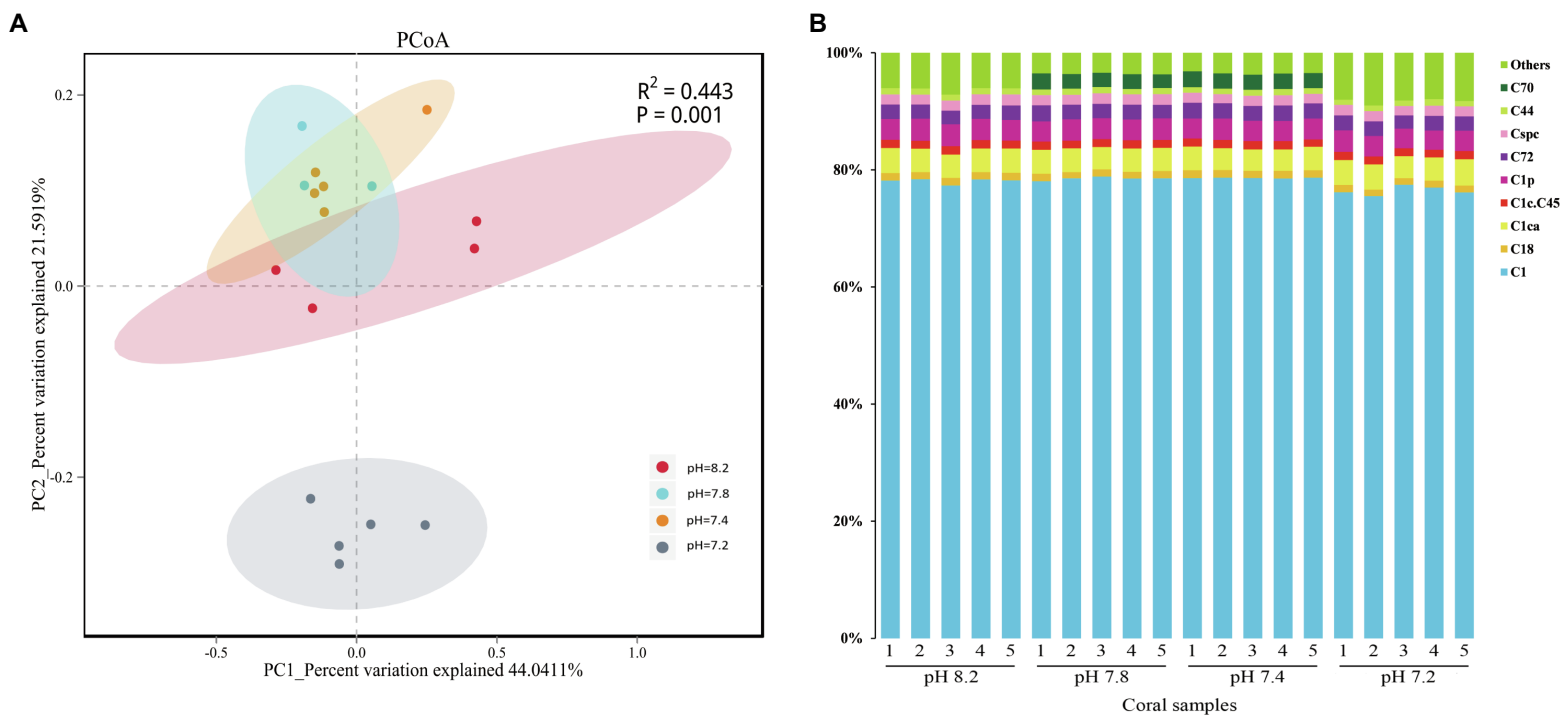
Similarity of community structure and relative abundance of Symbiodiniaceae. **(A)** Principal coordinates analysis (PCoA) on operational taxonomic unit (OTU) level. **(B)** Relative abundance of different Symbiodiniaceae subclades in coral samples.

The community composition of the Symbiodiniaceae in 20 coral samples was evaluated under four pH gradients (pH 8.2, 7.8, 7.4, and 7.2). The composition of the family group was analyzed by PCoA based on the OTU levels, wherein the first and the second principal components (PC1 and PC2), which accounted for 44 and 22% of the overall variance variation, showed the differences in the community composition of Symbiodiniaceae under different pH conditions (Figure 3A). In total, the community composition of the Symbiodiniaceae group had significant differences under the different pH gradients (PERMANOVA, *p* = 0.001).

The composition of Symbiodiniaceae subclades showed that *A. valida* is associated with many species of Symbiodiniaceae, including C1, C1ca, C1p, C72, Cspc, C1c.C45, C18, C18, C44, and C70 (Figure 3B), of which the C1 subclade had an absolute advantage. Overall, except for the presence of C70 in the dominant types at pH 7.8 and 7.4, the composition of the Symbiodiniaceae dominant types in *A. valida* was hardly changed during the entire acidification process. The difference analysis showed that the relative abundance of C70 increased significantly when the pH dropped from 8.2 to 7.8 (Kruskal-Wallis, C70: *H* = 16.269, *p* = 0.001, *post hoc*: Duncan), which increased from the background types to the dominant types. When the pH reached 7.4, the average relative abundance of C70 was 2.62%. When the pH dropped to 7.2, the relative abundance of C70 dropped below 1%, accounting for only 0.001%. Under the condition of pH 7.2, the relative abundance of C1 subclade dropped significantly to 76.44% (Kruskal-Wallis, C1: *H* = 14.657, *p* = 0.002, *post hoc*: Duncan), whereas those of the background types (Figure 3B: Others) rose significantly to 8.27% (Kruskal–Wallis, Others: *H* = 16.211, *p* = 0.001, *post hoc*: Duncan).

### Changes in the Community Composition of Coral-Associated Bacteria

**Figure 4:**
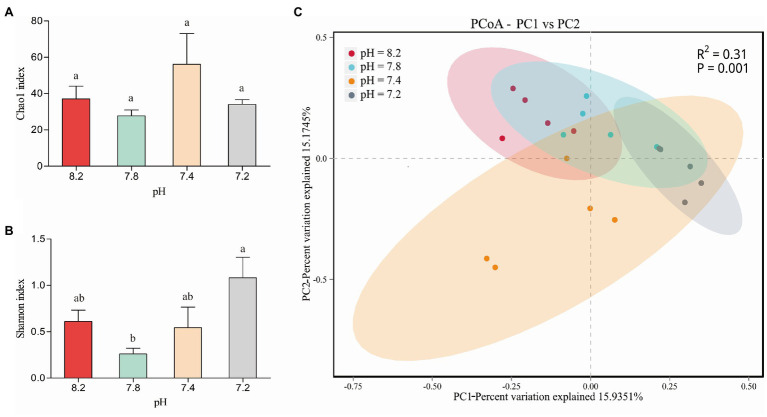
**(A)** Chao1 diversity and **(B)** Shannon diversity indices values of *Acropora valida*-associated bacteria between different pH. The error bars are the mean ± SD. values **(C)** PCoA of the unweighted UniFrac distance matrix representing differences in community composition at the OTU level. Lower case letters (a, b, c) denote significant differences by Duncan post hoc test.

We performed a coral-associated bacterial community composition analysis on the 20 samples under the four different pH gradients (pH 8.2, 7.8, 7.4, and 7.2). Twenty sample databases were obtained from the high-throughput sequencing for a total of 154,320 sequence numbers, and the sequence length was 1,000–1,800 bp. The number of sequences in each sample was more than 6,036, and the coverage index was greater than 99.8%, indicating that the sequencing coverage of each sample could genuinely reflect the actual situation of the coral-associated bacteria. Species annotation analysis detected a total of 10 bacterial phyla, 13 classes, 44 orders, 65 families, 86 genera, and 68 species, wherein the number of species was less than that of the genera because some species could be annotated to the genus level in the database, and there was no annotation information at the species level. Moreover, 165 OTU clusters were detected. Figures 4A,B show the changes in the alpha diversity indices of the four groups of coral samples. At the OTU level, the four pH gradients had no significant differences in the Chao1 index. Among them, the coral-associated bacterial community had the highest abundance at pH 7.4. A significant difference in the bacterial community diversity (Shannon) between pH 8.2 and 7.8 was observed (Student’s *t*, *p* < 0.05), and the differences in the Shannon indices between the pH 7.2 and 7.8 groups were also significant (Student’s *t*, *p* < 0.01). At pH 7.2, the bacteria community associated with *A. valida* had the highest diversity.

In the PCoA based on the OTU level, wherein PC1 accounted for 15.94% of the overall variance variation, the results showed the community composition of the coral-associated bacteria under the different pH conditions (Figure 4C). At pH 7.4, the degree of dispersion among the samples was the largest; that is, the difference between samples in this group was the largest. The two groups of pH 8.2 and 7.8 had the highest similarity regarding the composition of the coral-associated bacterial community. The differences in the coral-associated bacteria between the two groups of pH 8.2 and 7.2 were the largest, and the community composition changes between the groups were also the largest. Overall, the differences in the community composition of the coral-associated bacteria were significant under the different pH gradients (PERMANOVA, *p* = 0.001).

**Figure 5:**
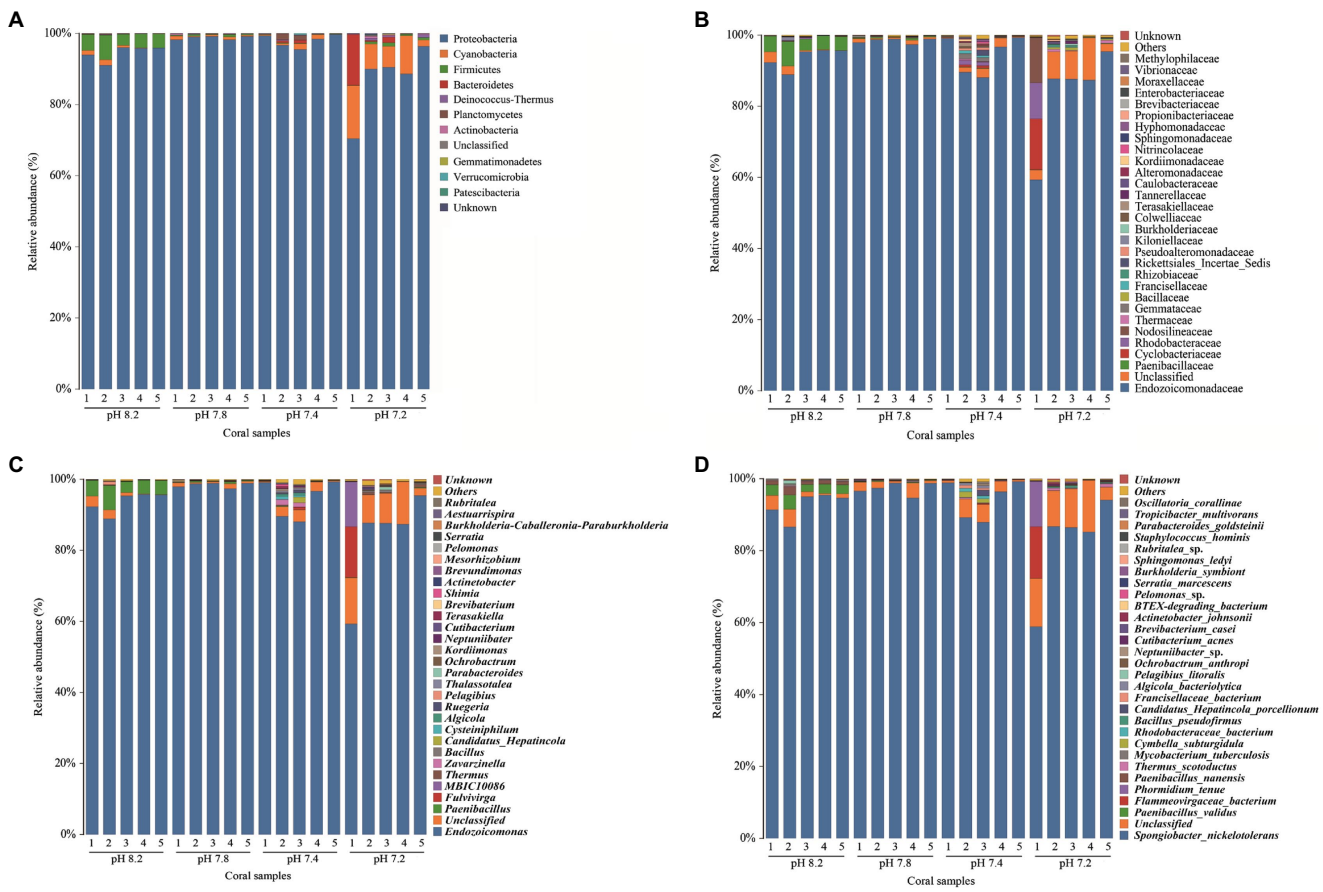
Relative abundance of bacteria at different taxonomic levels in each pH group. **(A)** phylum, **(B)** family, **(C)** genus, and **(D)** species level.

Figure 5A shows that the most abundant commensal bacterial phyla in *A. valida* mainly included Proteobacteria, Firmicutes, Cyanobacteria, Bacteroidetes, Deinococcus-Thermus, Planctomycetes, and Actinobacteria. During the entire acidification process, the commensal bacteria took Proteobacteria as the first dominant bacteria phyla. When the seawater pH was 8.2, 7.8, and 7.4, the relative abundance of Proteobacteria was 94.52, 98.77, and 97.91%, respectively. When the seawater pH dropped to 7.2, the average relative abundance of Proteobacteria dropped to 87.12% (Kruskal-Wallis: *H* = 13.057, *p* = 0.005, *post hoc*: Duncan). The relative abundance of Firmicutes at pH 8.2 was 4.51% ± 0.64%. However, when acidified to pH 7.8, the relative abundance of Firmicutes dropped rapidly and significantly below 1% (Kruskal-Wallis: *H* = 13.057, *p* = 0.005, *post hoc*: Duncan). It is worth noting that the relative abundance of Cyanobacteria significantly increased to 8.11 ± 0.23% at pH 7.2 (Kruskal-Wallis: *H* = 10.726, *p* = 0.013, *post hoc*: Duncan). Figure 5B shows the main components included Endozoicomonadaceae (Proteobacteria; Gammaproteobacteria; Oceanospirillales), Paenibacillaceae (Firmicutes; Bacilli; Caryophanales), Cyclobacteriaceae (Bacteroidetes; Proteophagia; Cytophagalobacteria), and Rhodobacteraceae (Proteobacteria; Alphaproteobacteria; Rhodobacterales) at the family level. At the genus level (Figure 5C), the community composition changes were consistent with that at the phylum level. *Endozoicomonas* (Proteobacteria; Endozoicomonadaceae) was the first dominant genus, wherein the relative abundance was between 58.70 and 99.31%. When the pH of the surrounding seawater dropped to 7.2, the relative abundance of *Endozoicomonas* decreased significantly (Kruskal-Wallis: *H* = 11.354, *p* = 0.010, *post hoc*: Duncan). Before the pH value of the surrounding seawater decreased (pH 8.2), *Paenibacillus* (Firmicutes; Bacillales) was the second most dominant genus, with a relative abundance of 4.48 ± 0.64%. After acidification stress, the relative abundance of *Paenibacillus* dropped significantly to 1% or less (Kruskal-Wallis: *H* = 14.885, *p* = 0.002, *post hoc*: Duncan). In addition, when the pH of the surrounding seawater dropped to 7.2, the proportion of unclassified bacteria increased significantly (Kruskal-Wallis: *H* = 8.646, *p* = 0.034, *post hoc*: Duncan), and the relative abundance proportion was 8.74 ± 1.93%. In general, compared with the two high pH groups (pH 8.2 and 7.8), when the surrounding seawater was acidified to a lower value (pH 7.4 and 7.2), the abundance of coral-associated bacteria increased significantly at the genus level. As shown in Figure 5D, the relative abundance of *Spongiobacter_nickelotoleran* (Proteobacteria; Oceanospirillales; *Endozoicomonas*) was the highest at the species level, fluctuating between 58.42 and 99.29%. When the pH of the surrounding seawater dropped to pH 7.2, the mean relative abundance of *Spongiobacter_nickelotoleran* was significant, dropping to 82.18 ± 6.1% (Kruskal-Wallis: *H* = 4.92, *p* = 0.178, *post hoc*: Duncan). Before the pH value of the surrounding seawater was lowered (pH 8.2), *Paenibacillus_validus* (Firmicutes; Bacillales; *Paenibacillus*) was the second most dominant species, with a relative abundance of 2.77 ± 0.32%. After the implementation of acidification, the relative abundance of *Paenibacillus_validus* dropped significantly to 1% or less (Kruskal-Wallis: *H* = 13.959, *p* = 0.003, *post hoc*: Duncan). In addition, when the pH of the surrounding seawater dropped to 7.2, the proportion of unclassified species increased significantly (Kruskal-Wallis: *H* = 8.189, *p* = 0.042, *post hoc*: Duncan), and the total relative abundance proportion was 10.43 ± 1.90%.

**Figure 6:**
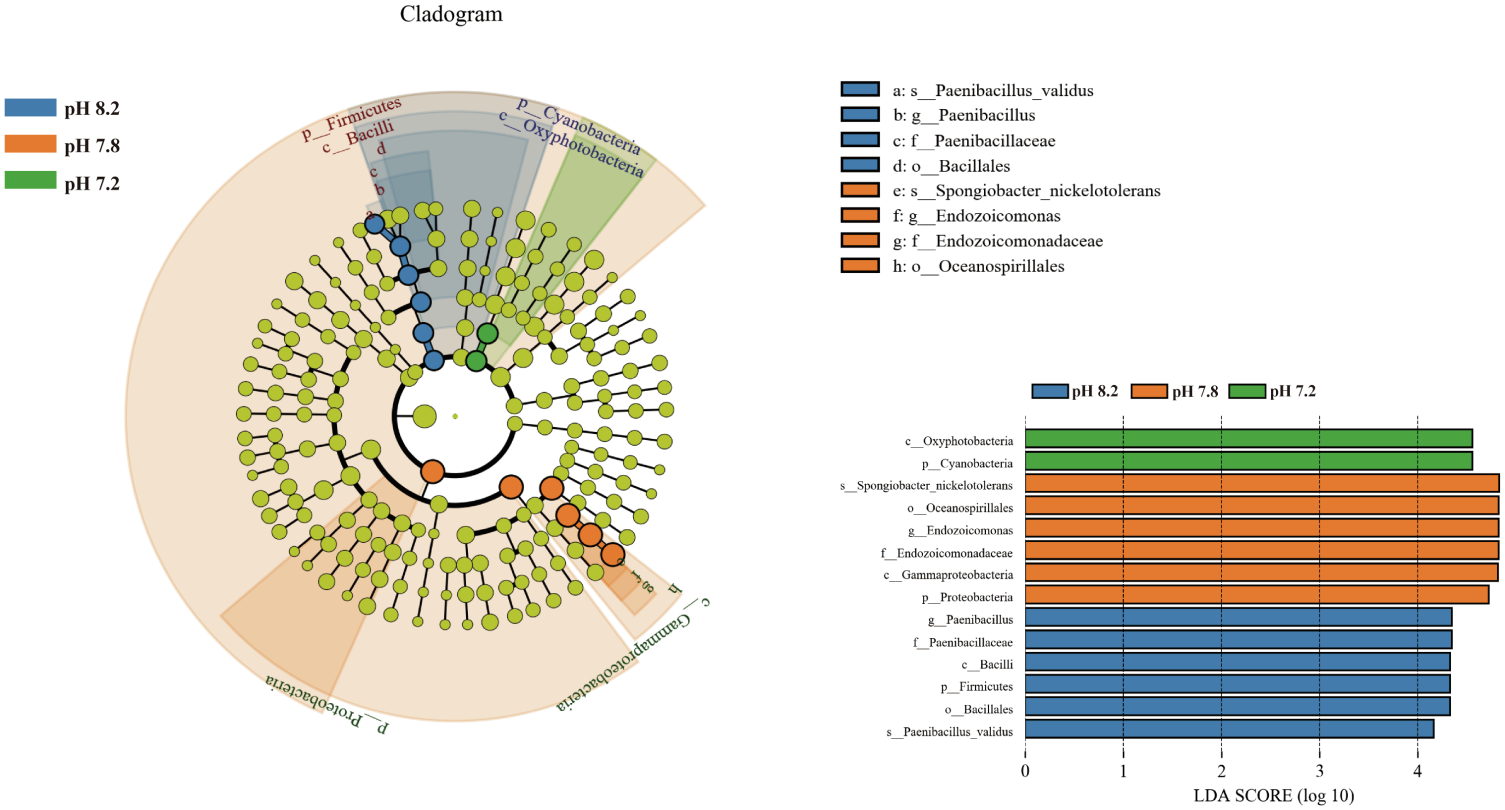
Bacterial communities with statistically significant differences. Cladogram displaying the different microbiota structures from phylum to genus level. Linear discriminant analysis score > 4. Different-colored regions represent different constituents (blue, pH 8.2; orange, pH 7.8; green, pH 7.2; and yellow: nonsignificant). The diameter of each circle is proportional to the abundance of the group.

Linear discriminant analysis effect size was used to analyze the significant differences between groups of bacterial communities from phylum to species (Figure 6). Fourteen taxa showed significant differences. Biomarkers with a pH 8.2 gradient belonged to Firmicutes, which were Firmicutes at the phylum level, Bacilli at the class level, and Bacillales at the mesh level. Paenibacillaceae at the family level, *Paenibacillus* at the genus level, and *Paenibacillus_validus* at the species level. Proteobacteria (Gammaproteobacteria, Oceanospirillales, Endozoicomonadaceae, *Endozoicomonas*, and *Spongiobacter_nickelotoleran*) under pH 7.8 showed significant differences from other pH groups; no significant biomarkers were found at the pH 7.4 gradient; and when the pH dropped to 7.2, the phylum level Cyanobacteria and Oxyphotobacteria at the class level become significant biomarkers under the acidification conditions.

## Discussion

### *Acropora valida* Shows Strong Adaptability to Ocean Acidification

During the acidification simulation experiment, when the pH value of the surrounding seawater continued to decrease, *A. valida* did not experience apparent bleaching, and the coral tentacles were still able to stretch freely (Figure 1). The results showed that the Symbiodiniaceae density decreased significantly under ocean acidification (Figure 2A). When the pH dropped to 7.8 or below, the Symbiodiniaceae density decreased significantly. When the pH reached 7.2, the Symbiodiniaceae density was 0.22 ± 0.16 × 10^6^ cells·cm^−2^, which was about 19% of the density at pH 8.2. It is noteworthy that, despite the continuous decrease of Symbiodiniaceae density under ocean acidification, the real-time photosynthetic efficiency of *A. valida* did not change significantly and remained at about 0.62 μmol·m^2^·s^−1^ (Figure 2B).

Although there have been previous reports on coral bleaching caused by acidification (Anlauf et al., 2011), more and more pieces of evidence show that many corals have strong adaptability to acidification (Schneider and Erez, 2006; Anlauf et al., 2011; Baghdasarian et al., 2017; Krueger et al., 2017; Meunier et al., 2021; Zhou et al., 2021). For example, Noonan and Fabricius (2015) studied the response of *Seriatopora hystrix* and *Acropora millepora* to ocean acidification, finding that elevated CO_2_ partial pressure (pH 7.79) had no adverse effects on both coral (Noonan and Fabricius, 2015). Moreover, Wall et al. (2014) found that elevated CO_2_ concentration did not affect the photosynthetic physiological parameters and productivity of *Seriatopora caliendrum* holobiont nor did it cause coral bleaching (Wall et al., 2014), whereas Krueger et al. (2017) found that *Stylophora pistillata* on Aqaba Island did not show bleaching after 1.5 months under high temperature and acidification (pH 7.8; Krueger et al., 2017). Furthermore, Rodolfo-Metalpa et al. (2010) found that acidification (pCO_2_ = 700 μatm) did not affect the photosynthetic efficiency and calcification rate of *Cladocora caespitosa* nor did it cause coral bleaching (Rodolfo-Metalpa et al., 2010).

The Symbiodiniaceae density is an essential indicator of coral health (Jones and Yellowlees, 1997; Baker et al., 2008; Qin et al., 2019). Studies indicate that Symbiodiniaceae density would decrease with ocean acidification (Kaniewska et al., 2012; Mason, 2018). For example, Krief et al. (2010) found that the Symbiodiniaceae densities of *Porites* sp. and *S. pistillata* at pH 7.49 and 7.19 were significantly lower than that at pH 8.09 (Krief et al., 2010). Kaniewska et al. (2012) found that *A. millepora* lost more than 50% of the Symbiodiniaceae under high CO_2_ partial pressure (Kaniewska et al., 2012). However, it is noteworthy that the decrease in Symbiodiniaceae density does not necessarily cause coral bleaching or even death. Studies have shown that even healthy corals of the same species in the same sea area will show significant differences in the density of Symbiodiniaceae due to the alternation of the seasons; that is, there is no bleaching in the summer and autumn when the density of Symbiodiniaceae is at its lowest (Shenkar et al., 2006; Li et al., 2008; Xu et al., 2017). Li et al. (2008) studied the effects of high temperature on *Pocillopora damicornis* in the Hainan Island and found that 32°C high-temperature stress caused the loss of 43.5% of the Symbiodiniaceae in the *P. damicornis*, but this did not lead to coral bleaching (Shu et al., 2008). Moreover, studies on the density of Symbiodiniaceae in corals in the southern South China Sea also showed that even though the density of Symbiodiniaceae in corals was 31–53% lower than that in the other areas, the corals still survived and maintained good coverage without bleaching (Li et al., 2011).

The photosynthetic efficiency of coral symbiotic algae can also indicate coral health (Smith et al., 2005; Xu et al., 2017). Research by Mason (2018) showed that the *Galaxea fascicularis* excreted 71% of the Symbiodiniaceae under acidification. However, the remaining Symbiodiniaceae increased their photosynthetic pigments to maintain the energy supply through photosynthesis (Mason, 2018). Schneider and Erez (2006) reported that *A. eurystoma* photosynthetic efficiency did not change significantly under pH 7.9–8.5 (Schneider and Erez, 2006). Furthermore, Zheng et al. (2015) believed that ocean acidification (pH 7.8) had no significant effect on the maximum photosynthetic efficiency of *P. damicornis* (Zheng et al., 2015), and Wall et al. (2014) believed that the effect of acidification on the photosynthetic efficiency of *S. caliendrum* is negligible (Wall et al., 2014). Some studies even show that acidification is beneficial to the photosynthesis of coral symbiotic algae (Pearse and Muscatine, 1971; Crawley et al., 2010). For example, Uthicke and Fabricius (2012) found that with the increase of carbon content in seawater (pH 7.8), the primary photosynthetic productivity of *Marginopora vertebralis* symbiotic algae increased by 90% (Uthicke and Fabricius, 2012). Biscéré et al. (2019) found that rates of photosynthesis by Symbiodiniaceae were higher at the high CO_2_ site compared to the ambient site in Papua New Guinea (Biscéré et al., 2019).

In this study, although acidification reduced the Symbiodiniaceae density of *A. valida* by about 80%, acidification had almost no noticeable effect on its photosynthetic efficiency, and the coral tentacles were still able to stretch freely. Taken together, this study demonstrated that *A. valida* is very adaptable to ocean acidification.

### The Community Composition of the Coral-Symbiodiniaceae Symbiosis System Is Hardly Affected by Acidification

The composition of the dominant types of Symbiodiniaceae of *A. valida* did not change significantly during the entire acidification process, and the C1 subclade always occupied an absolute advantage, with a stable symbiosis with *A. valida* during the entire acidification process (Figure 3B). However, at pH 7.2, the relative abundance of the Symbiodiniaceae background types (Figure 3B, Others) increased significantly to 8.27%.

Several studies currently suggest that ocean acidification has no significant impact on the dominant types of Symbiodiniaceae (Noonan et al., 2013; Davies et al., 2018). For example, Zhou et al. (2017) studied the Symbiodiaceae community of *A. gemmifera* under different pH conditions (pH 8.1, 7.8, and 7.5) and found that it was always dominated by the D17 group (Zhou et al., 2017). Davies et al. (2018) also found that acidification (pCO_2_ = 2,553 μatm) had no significant effect on the dominant types of Symbiodiniaceae (C1 subclade) in *Siderastrea sidereal* (Davies et al., 2018). Moreover, the Symbiodiniaceae composition of six coral species in the CO_2_ crater (pH 7.8–7.9) of PNG and of the same corals far away from the crater (pH 8.0–8.05) were found to have no difference (Noonan et al., 2013). The above results are consistent with the experimental results of the present study. The C1 subclade had high photosynthetic efficiency and tolerance to low temperature and nitrogen-rich environments (Baker et al., 2013; Ng and Ang, 2016; Morgans et al., 2020). Previously, the D clade Symbiodiniaceae were considered to have stronger high-temperature resistance (Berkelmans and van Oppen, 2006; LaJeunesse et al., 2014). However, the latest research shows that *Cladocopium goreaui* (C1) and *Durusdinium trenchii* (D1a) are used as probiotics and have been inoculated into *A. millepora* and subjected to heat stress. Corals with *C. goreaui* (C1) showed less bleaching and had an increased photosynthetic efficiency (Morgans et al., 2020). Cantin et al. (2009) found that in *A. millepora* Symbiodiniaceae, the relative electron transport of the C1 subclade through photosystem II was 87% higher than that of the D clade (Cantin et al., 2009). Given that the C1 subclade is a type of Symbiodiniaceae with high photosynthetic capacity, it is reasonable to speculate that the dominant advantage of the C1 subclade in *A. valida* may explain the stability of photosynthetic efficiency in the acidification experiment and compensate for the adverse effects of acidification on the Symbiodiniaceae density.

The Symbiodiniaceae background types serve as the basis for the plasticity of Symbiodiniaceae communities (Shade et al., 2014), which stability is affected by environmental changes (Quigley et al., 2014). Ziegler et al. (2018) reported that the change in the dominant types could not explain the range of coral response to pressure because the dominant types share the coral reef ecosystem in a broad environmental gradient, and the members of the Symbiodiniaceae background types are likely to be the same as the members of the rare bacterial biosphere, which are more active than the members of the dominant group; therefore, different members of the background types may be an essential part of supporting corals in response to environmental changes (Ziegler et al., 2018). When environmental stress induces reorganization or conversion of Symbiodiniaceae, the interaction between members of the Symbiodiniaceae background types may be more prominent (Boulotte et al., 2016). A theoretical network modeling on the relationship between corals and Symbiodiniaceae under climate change predicts that the increase in Symbiodiniaceae diversity and the decrease in Symbiodiniaceae types often occur at low abundance (Fabina et al., 2013). They provide redundancy or complementary symbiotic functions that can significantly improve the stability of the response of the community to environmental changes. Therefore, although the Symbiodiniaceae background types in this acidification experiment did not account for a high proportion, the significant increase in the relative abundance of the Symbiodiniaceae background types at pH 7.2 indicates that *A. valida* is likely to improve the stability of the Symbiodiniaceae community to acidification by increasing the abundance of the Symbiodiniaceae background types.

In summary, this short term of ocean acidification did not have a significant impact on the composition of the Symbiodiniaceae dominant types in *A. valida*. The stable symbiosis of the C1 subclade with high photosynthetic capacity and *A. valida* probably compensated for the adverse effect of acidification on the Symbiodiniaceae density, maintaining the stability of photosynthetic efficiency. The significant increase in the relative abundance of the *A. valida* Symbiodiniaceae background types is likely to enhance the stability of the Symbiodiniaceae community and help corals adapt to the acidic environment. However, the changes of the Symbiodiniaceae community composition in *A. valida* under long-term acidification stress are worthy of further study.

### Changes in the Composition of the Coral-Associated Bacteria Help Corals Adapt to an Acidified Environment

Using the *16S* rRNA clone library to identify and analyze the microbial community of *A. valida*, four major bacterial phyla were identified – Proteobacteria, Firmicutes, Cyanobacteria, and Bacteroidetes (Figure 5A) – that agree with previous findings (Bourne and Munn, 2005; Sekar et al., 2006; Wegley et al., 2007). At the genus level, the coral probiotic *Endozoicomonas* always dominated (Figure 5C). In addition, at pH 7.8, the relative abundance of Firmicutes decreased significantly, whereas, at pH 7.2, the relative abundance of Cyanobacteria increased significantly (Figure 5A).

*Endozoicomonas* (Gammaproteobacteria) is a bacterium that exists widely in coral reefs globally (Neave et al., 2016) because it accounts for a relatively high proportion of healthy corals (Vezzulli et al., 2013; O’Brien et al., 2016; Shiu et al., 2017) and is thus considered as a potential indicator of coral health (Bourne et al., 2008). Studies based on catalytic messenger deposition-fluorescence *in situ* hybridization (CARD-FISH) have proved that *Endozoicomonas* symbiotically exists in the endoderm tissue of corals and are closely related to corals (Bayer et al., 2013; Neave et al., 2017). *Endozoicomonas* belongs to Family Hahellaceae and Order Oceanospirillales and is a group of heterotrophic, aerobic marine bacteria that can decompose various organic compounds (Garrity, 2007). Studies have shown that the main function of *Endozoicomonas* is to participate in the sulfur cycle of the coral holobiont by metabolizing dimethylsulfoniopropionate (DMSP; Raina et al., 2009, 2010). It provides beneficial protection for the Symbiodiniaceae in the holobiont from albino pathogens (Morrow et al., 2012; Pantos et al., 2015), participates in the transport of protein and carbohydrates (La Riviere et al., 2013), and provides advantages for coral health by producing antibacterial compounds (Bourne et al., 2008). Therefore, the absolute advantage of *Endozoicomonas* is likely to help *A. valida* adapt to ocean acidification.

The adaptation of *A. valida* to ocean acidification may also be closely related to changes in the composition of the coral-associated bacterial community. Meron et al. (2011) found that the relative abundances of Firmicutes, Bacteroidetes, and Cyanobacteria in *A. millepora* increased under acidification (pH 7.3). In addition, the relative abundances of *Vibrio* sp. and *Alteromonas* sp. related to diseases in *A. millepora* were increased. It also increased significantly (Meron et al., 2011). Some studies suggest that Bacteroidetes are related to the Black Band Disease and that Firmicutes and Bacteroidetes come from the same diseased tissue (Cooney et al., 2002; Frias-Lopez et al., 2002, 2004). Therefore, the increase in Firmicutes and Bacteroidetes may be a manifestation of the threat of acidification to coral health (Meron et al., 2011). The present experiments did not find an increase in disease-related Bacteroidetes, Firmicutes, *Vibrio* sp., and *Alteromonas* sp., and the relative abundance of Firmicutes also decreased significantly at pH 7.8, indicating that *A. valida* was in a healthy state throughout the acidification experiment. Being in a healthy state during coercion is also consistent with the appearance of *A. valida* without bleaching or disease. As important photosynthetic microorganisms in seawater, Cyanobacteria are closely related to the nitrogen fixation that depends on photosynthesis in coral reefs (Lesser et al., 2004). The increase in Cyanobacteria contributes to the increase in carbon fixation capacity (Zhang et al., 2018). Studies have shown that some reef sponges can increase the relative abundance of Cyanobacteria to make them more competitive than corals under ocean acidification (Bell et al., 2013; Morrow et al., 2015). Therefore, the increase in Cyanobacteria under acidification stress (pH 7.2) may be a response of *A. valida* to the composition of bacterial communities to adapt to acidification stress. The increase in Cyanobacteria is likely to provide compensation for the photosynthesis of coral holobiont, ensuring the energy supply that is needed by corals in low pH environments. A new study by Meunier et al. (2021) found that at the CO_2_ seeps of Tutum Bay (PNG), *P. damicornis* modified its N assimilation pathways by utilizing picoplankton-sized unicellular Cyanobacteria (*Synechococcus* sp.) in oligotrophic waters to offset the increasing N requirements under high CO_2_ (Meunier et al., 2021). This is consistent with our research results.

In summary, the stable symbiosis of *Endozoicomonas* and *A. valida* under acidification stress may help it adapt to the acidified environment. The increase in the relative abundance of Cyanobacteria and other photosynthetic bacteria at pH 7.2 is likely to contribute to the photosynthesis of coral symbionts and ensure the energy supply for coral survival in a low pH environment. Therefore, the stability and flexibility of the coral-associated bacterial community help *A. valida* to withstand acidification stress.

## Conclusion

In this study, an indoor simulation of ocean acidification was conducted. Changes in the composition of the Symbiodiniaceae and coral-associated bacterial communities of reef-building corals under different pH degrees occurred. Hence, the response of *A. valida* to ocean acidification is likely a complex biological process, which includes the dynamic changes in the coral hosts, coral-associated bacteria, and Symbiodiniaceae. This study suggests that, although ocean acidification caused a significant decrease in the density of Symbiodiniaceae, *A. valida* did not show significant bleaching, with the real-time photosynthetic efficiency of the holobiont remained unchanged and the coral tentacles were still able to stretch freely, which well-adapted to the acidification stress. Thus, the Symbiodiniaceae and bacteria of the coral holobiont were involved in corals adapting to acidification stress. Regarding the Symbiodiniaceae, the stable symbiosis between the C1 subclade, with the high photosynthetic ability, and *A. valida* in the dominant system group probably compensated for the negative impact of acidification on the density of Symbiodiniaceae, explaining why the photosynthetic efficiency remained stable. In addition, the increase in the relative abundance of the Symbiodiniaceae background types could enhance the stability of the Symbiodiniaceae community composition. In terms of the coral-associated bacteria, the stable symbiosis of *Endozoicomonas* and *A. valida* under acidification stress could help *A. valida* to adapt to the acidification environment, and the significant increase in the relative abundance of Cyanobacteria at pH 7.2 could provide compensation for the photosynthetic efficiency of the coral symbiosis. In summary, the stability and flexibility of the Symbiodiniaceae community and the coral-associated bacterial community together helped *A. valida* resist the threats of ocean acidification, which also indicates that corals can adapt to ocean acidification by adjusting their holobiont.

## Data Availability Statement

The data presented in the study are deposited in the NCBI SRA repository. The accession numbers are PRJNA761014 for the sequence of *Acropora valida*-associated bacteria and PRJNA761031 for the sequence *Acropora valida* symbiotic Symbiodiniaceae respectively.

## Author Contributions

RG: conceptualization, methodology, formal analysis, investigation, resources, project administration, data curation, writing – original draft, writing – review and editing, visualization, software, and project administration. JL: conceptualization, methodology, validation, writing – review and editing, and supervision. KY: conceptualization, writing – review and editing, supervision, and funding acquisition. BC: software and writing – review and editing. XY: writing – review and editing. CD, JC, YX, and LQ: resources. All authors contributed to the article and approved the submitted version.

## Funding

This work was supported by the National Natural Science Foundation of China (Nos. 42030502 and 42090041), the Guangxi scientific projects (No. AD17129063 and AA17204074), the BaGui Scholars Program Foundation (No. 2014BGXZGX03), and the Natural Sciences Foundation of Guangxi (2018GXNSFAA281328).

## Conflict of Interest

The authors declare that the research was conducted in the absence of any commercial or financial relationships that could be construed as a potential conflict of interest.

## Publisher’s Note

All claims expressed in this article are solely those of the authors and do not necessarily represent those of their affiliated organizations, or those of the publisher, the editors and the reviewers. Any product that may be evaluated in this article, or claim that may be made by its manufacturer, is not guaranteed or endorsed by the publisher.
